# Spatiotemporal Analysis of COVID-19 Incidence Data

**DOI:** 10.3390/v13030463

**Published:** 2021-03-11

**Authors:** Ilaria Spassiani, Giovanni Sebastiani, Giorgio Palù

**Affiliations:** 1 Istituto Nazionale di Geofisica e Vulcanologia, Via di Vigna Murata 605, 00143 Rome, Italy; ilaria.spassiani@ingv.it; 2 Istituto per le Applicazioni del Calcolo Mauro Picone, Consiglio Nazionale delle Ricerche, Via dei Taurini 19, 00185 Rome, Italy; 3 Mathematics Department “Guido Castelnuovo”, Sapienza University of Rome, Piazzale Aldo Moro 5, 00185 Rome, Italy; 4 Department of Mathematics and Statistics, University of Tromsø, H. Hansens veg 18, 9019 Tromsø, Norway; 5 Department of Molecular Medicine, University of Padua, Via Gabelli 63, 35121 Padua, Italy; giorgio.palu@unipd.it

**Keywords:** COVID-19, mathematical analysis, spatial distribution, hierarchical clustering, networks

## Abstract

(1) Background: A better understanding of COVID-19 dynamics in terms of interactions among individuals would be of paramount importance to increase the effectiveness of containment measures. Despite this, the research lacks spatiotemporal statistical and mathematical analysis based on large datasets. We describe a novel methodology to extract useful spatiotemporal information from COVID-19 pandemic data. (2) Methods: We perform specific analyses based on mathematical and statistical tools, like mathematical morphology, hierarchical clustering, parametric data modeling and non-parametric statistics. These analyses are here applied to the large dataset consisting of about 19,000 COVID-19 patients in the Veneto region (Italy) during the entire Italian national lockdown. (3) Results: We estimate the COVID-19 cumulative incidence spatial distribution, significantly reducing image noise. We identify four clusters of connected provinces based on the temporal evolution of the incidence. Surprisingly, while one cluster consists of three neighboring provinces, another one contains two provinces more than 210 km apart by highway. The survival function of the local spatial incidence values is modeled here by a tapered Pareto model, also used in other applied fields like seismology and economy in connection to networks. Model’s parameters could be relevant to describe quantitatively the epidemic. (4) Conclusion: The proposed methodology can be applied to a general situation, potentially helping to adopt strategic decisions such as the restriction of mobility and gatherings.

## 1. Introduction

A new coronavirus, causing a severe acute respiratory syndrome (COVID-19), and being transmitted between individuals, was originally identified as 2019nCoV in Wuhan (China) in December 2019 and subsequently named SARS-CoV-2 for its 80% genome homology to that of the HCoV SARS (SARS-CoV-1) and the resemblance of its clinical manifestations to those of the aforementioned virus [1,2,3]. The epidemic was rapidly spreading from China throughout the world, to become a pandemic that, as of today, has affected more than 118 million people, causing over 2.6 million deaths [4,5]. During the first phase of massive campaign of vaccination to prevent SARS-CoV-2 infection and the related disease COVID-19, we still need to use the common measures to attempt containing pandemic such as facial masks and disinfectants, avoiding people gathering and social distancing, massive testing and tracing, or more severe restrictions like quarantine, curfew or lockdowns of all near-contact activities. A better understanding of SARS-CoV-2 dynamics would be of great importance, in order to increase the effectiveness of the above containment measures, giving the health authorities reliable means to curb virus spreading in their territories, by scaling up adequate actions. Therefore, statistical and mathematical models are needed, that would be able to predict pandemic evolution and emergency management with related social and medical impact. These analyses of the pandemic data could allow to study the influence of some relevant factors like the interaction among individuals on the incidence distribution and spatiotemporal pattern.

The spatial analysis of COVID-19 has been reaching a growing interest and includes health and social geography, environmental variables, data mining, web-based mapping and space-time statistical analysis, and our paper belongs to the latter category, where works at different spatial scales (e.g., provinces or nations) for many countries worldwide have been published, see [6] and references therein. In a recent paper [7], the authors propose to use a multivariate time-series mixed-effects generalized linear model for describing the spatiotemporal evolution of the COVID-19 pandemic. They apply it to provide reliable predictions of infectious diseases in time and space for areal disease in Italy. In particular, they describe the expected number of COVID-19 infections by three subcomponents: the epidemic-within (intra-provinces), the epidemic-between (inter-provinces), and the endemic (due to a priori province-specific conditions, independently of the epidemic process). From the incidence curves at the province level, they compute these three components for the provinces in Italy, and obtain three maps for the Italian territory. As we are going to see, one of the approaches that we consider, involving clustering, is not coped in that paper. Other methodologies typically used in the literature to depict space-time patterns of SARS-CoV-2 and COVID-19 are based on Moran’s *I* measure and the local indicators of spatial association (LISA) statistic to analyze the global and local clusters as well as spatial outliers—see, for example, [8,9,10]. In particular, the LISA map shows four categories of spatial patterns: the high-high and low-low locations (positive local spatial autocorrelation) were typically referred to as spatial clusters, while the high-low and low-high locations (negative local spatial autocorrelation) were termed spatial outliers. These are both non-parametric methodologies, helpful in their ease and broad applicability. The approach that we consider here is instead based on parametric modeling, which is less general, but more robust in the specific case we are considering. In fact, here, we are able to identify a suitable parametric model with a good fit for the daily incidence data that we analyze.

In this paper, we aim at studying the spatiotemporal dynamics of the SARS-CoV-2 pandemic from data of COVID-19 patients, as we think its results could be very relevant in helping decision makers to plan community actions for facing and overcoming infection. In particular, we cope with the important issue of partitioning a spatial region of interest—e.g., the whole country, a region, a province, a municipality—into separated components of sub-units sharing a common pattern of diffusion. This information could be very useful to identify the areas where more restrictive measures should be implemented to limit the pandemic diffusion, minimizing, at the same time, the flux of people across the boundaries of each area. We also aim at providing a way to quantitatively describe the diffusion through a function of positive cases’ incidence, strongly related to the network of the infection contacts, to be used together with the standard parameters typically adopted to quantify the pandemic diffusion, like for example the effective and very commonly used reproduction number, which is based on first symptoms incidence.

We use mathematical and statistical tools like mathematical morphology, hierarchical clustering, parametric fitting and non-parametric hypothesis testing. We apply the developed methodologies to the data in the Veneto region (Italy) during the Italian lockdown, at the national level. The cumulative COVID-19 incidence portrayed in a spatial configuration results in a less noisy image than the one directly calculated from data, which still contains most of the relevant spatial information. Based on the population normalized incidence curves of the seven provinces in Veneto, we identify four clusters of provinces, the elements of each cluster sharing similar patterns. The same four groups reflect similarities in the population density, which seems to be linearly related to the maximum normalized incidence value. Finally, the municipalities incidence’s survival function shows a well-known pattern, associated to the presence of an underlying network already found in different applied fields like seismology and economy.

## 2. Materials and Methods

The different analyses that we conduct are based on robust mathematical and statistical methods and involve mathematical morphology, hierarchical clustering, parametric data fitting and non-parametric hypothesis testing. The methods are implemented by us in Matlab language.

Let us consider the smallest rectangle containing the Veneto region (Italy). Since the data provided to us contain spatial information at the finest level of 1 km, we discretize this rectangle into pixels of size 1 × 1 km. We then first focus on the total number of patients positive to SARS-CoV-2 during the period from 12 March to 15 May 2020, for each pixel of this rectangle. We shall refer to it as incidence image. In order to reduce the perturbation in the incidence image in Veneto, here we apply the mathematical morphology opening operator, which consists in the application in turns of the basic operators: dilation and erosion [11]. Once we binarize (0–1) the image to be processed depending on their positive or null values, we fix a characteristic element (CE), i.e., a symmetric geometrical object (in our case, a cross of length 3 pixels). Then, we move this CE along all the pixels of the original image. The dilation image consists of all those pixels which are centers of CE containing at least a non-null value pixel of the binarized image. The erosion image consists instead of all those pixels which are centers of CE containing all non-null value pixels of the binarized image.

For all of the seven provinces in the Veneto region, we compute the total count $C_{i}$ of positive patients registered at each of the N days $\left\{t_{1},\;\ldots ,t_{N}\right\}$ in the considered temporal interval. We shall refer to this as the daily incidence sequence, which we model by means of the derivative of the following well-known logistic model [12], extended here with an additional parameter as an exponent of the denominator:

$${\hat{C}}_{i}=\frac{\beta_{1}}{{\left(1+\beta_{3}\;*exp\left\{-t_{i}/\beta_{2}\right\}\right)}^{\beta_{4}}},$$

where all parameters are positive. As commonly done, the parameters are obtained by means of the least squares criterion [13], which consists of minimizing the sum of squares of the deviations between data and model:

$$SSD=\overset{N}{\underset{i=1}{\sum }}{\left(C_{i}-{\hat{C}}_{i}\right)}^{2}.$$

Aiming at grouping Veneto provinces according to some relevant parameters of the model for their incidence curves, we perform the so-called hierarchical clustering algorithm [14,15]. This algorithm iteratively aggregates sample elements and clusters by minimizing locally the sum of squares of Euclidian distance between cluster elements and the corresponding centroid. Apart from a numerical constant, this is the within variance of the cluster. The output obtained is the aggregation tree known as dendrogram. After choosing a number of clusters k between 1 and the number of data points n, the associated clusters with their elements are obtained by suitably cutting the dendrogram with a horizontal line. Given an arbitrary number of clusters between 1 and n − 1, the corresponding number appearing on the vertical axis, divided by √2, gives the square root of the difference between the summation over the k clusters of the within cluster sum of squares and the same quantity for k + 1 clusters. To select the optimal number of clusters, we use these heights. Specifically, we follow the elbow criterion [16], slightly modified: we select the number k such that if we decrease it by one unit we have a high loss, while if we increase it by one unit, we have a low gain. More precisely, we maximize the ratio loss/gain, which indeed corresponds to the ratio between the heights relative to k − 1 and k clusters, respectively.

To verify the reliability of the results obtained by the clustering analysis, we compare the incidence mean values for the municipalities of different provinces. To do that, we perform the non-parametric Wilcoxon-Mann-Whitney hypothesis test [17], which is based on the sum of ranks of data points after their ascending ordering and consequent renumbering.

Finally, the survival function of the municipalities’ incidence is modeled by the well-known tapered Pareto law [18]. This model initially decreases as a power law function of the variable considered (here the incidence), then after a while, the model progressively decreases faster than the power law.

## 3. Results

We analyze the data relative to patients who tested positive for SARS-CoV-2. Oropharyngeal and nasal swabs were executed by trained personnel according to the Italian guidelines licensed by Istituto Superiore di Sanità (ISS). Specimens were immediately processed by means of multiplex realtime RT-PCR. Two technologies were used for SARS-CoV-2 gene target amplification, Seegene and Roche (CE-IVD certificate). The data were provided by UOC Sistemi Informativi Azienda Zero-Veneto Region (Padua, Italy), in anonymous aggregate form, in terms of a three-dimensional matrix representing the number of positive cases in all 1 km square cells covering the whole Veneto region, at each day in the interval from 12 March to 15 May 2020. The dataset is relative to about 270,000 SARS-CoV-2 tested subjects; 19,000 tested positive. It contains information about several relevant parameters of the patients, e.g., comorbidities, age and gender, some of which are already analyzed by us without focusing on spatial aspects [19]. The total volume of the dataset is 71.5 MB.

The very first quantity that we consider in our spatial analysis is the population density, that is the ratio between population and area (in sq. km) of the region considered. Based on these data, we notice that four density-based groups of provinces appear: the one corresponding to the lowest densities contains Belluno (55.9) and Rovigo (128), while the one with the highest density consists of Padua (438). The two other groups are Verona-Vicenza (300–317), and Treviso-Venice (358–344), with intermediate densities with inter-variability larger than the intra-variability.

**Figure 1:**
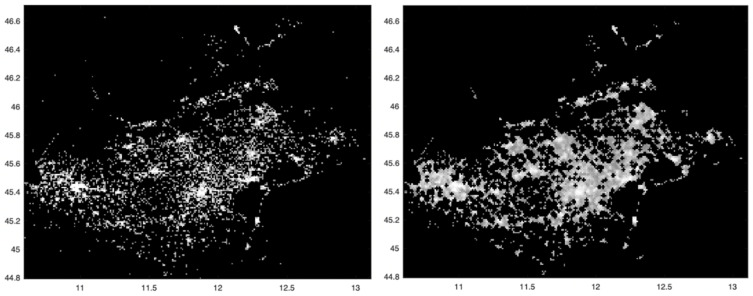
Left: spatial distribution of the SARS-CoV-2 incidence in the period from 12 March to 15 May 2020, in all 1 km squared cells covering the Veneto region. Darker pixels correspond to lower intensity values. To increase the contrast of the image, a non-linear mapping (fourth root) is applied. Outlier values with very high intensity were reassigned through the 0.993 quantile (low-pass filter). Right: mathematical morphology opening operator applied to the image in the left panel followed by removal of “isolated” points. The values along x and y axes refer respectively to longitude and latitude.

We now analyze the total number of SARS-CoV-2 positive cases at the finest spatial scale allowed by our data, that is, 1 squared km, as shown in Figure 1, left panel; a higher intensity corresponds to a higher number of cases. The three highest intensity spots can be observed in correspondence of the main municipality of the provinces of Verona, Padua and Venice, where the virus spread significantly. To follow, the main municipality of the provinces of Treviso and Vicenza show a slightly smaller intensity, while the total number of cases was significantly lower for the provinces of Rovigo and Belluno.

With the aim at trying to reduce the presence of noise in the incidence map of Figure 1, left panel, we now apply the mathematical morphology opening operator, followed by the removal of “isolated” points, i.e., those whose 24 neighboring pixels have zero incidence. The result is shown in the right panel of Figure 1. Compared to the left one, the structures appearing in the map are now more connected.

**Figure 2:**
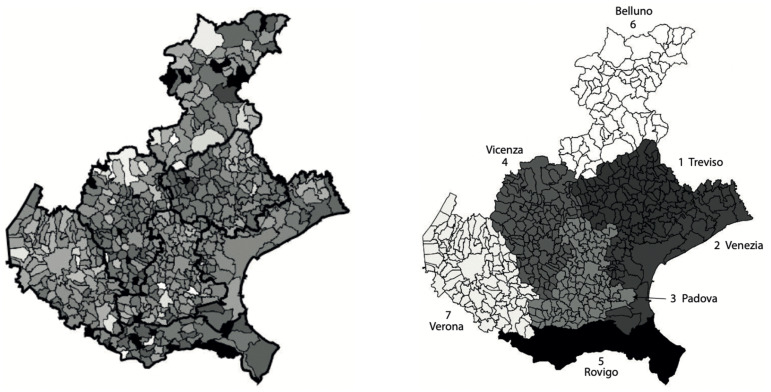
Left: map of the SARS-CoV-2 municipalities incidences in Veneto relative to the period from 12 March to 15 May 2020, normalized by the corresponding populations at 31 December 2019. Intensity increases with respect to the normalized incidence. The intensities of a few municipalities with outlier values of the incidence were rescaled to increase the contrast of the map. The provinces boarders are thicker. Right: map of the Veneto provinces normalized incidences, obtained as the ratio between the sum of the total number of cases in all province municipalities and the corresponding total province population. The populations for the provinces (numbered from 1 to 7) are, respectively: 888,309; 851,663; 939,672; 862,363; 233,386; 201,972; 930,339.

In Figure 2, left panel, we show the incidence of the municipalities normalized with respect to the corresponding population size (higher intensity for higher incidence). It can be observed that the province of Verona appears separated from the neighbor provinces, as its municipalities are lighter. A similar level of incidence is instead observable for the four provinces of Venice, Vicenza, Treviso and Padua, the municipalities of the last province showing a slightly lighter intensity. The average incidence of the province of Rovigo appears to be the lowest one, while the province of Belluno is the one showing the largest variability of the incidence. These results can be better appreciated in the right panel of Figure 2, where the average incidence over each of the seven provinces is shown. Here, the average incidence is calculated by dividing the sum of the total number of cases in all municipalities of each province, by the total population of the province.

**Figure 3:**
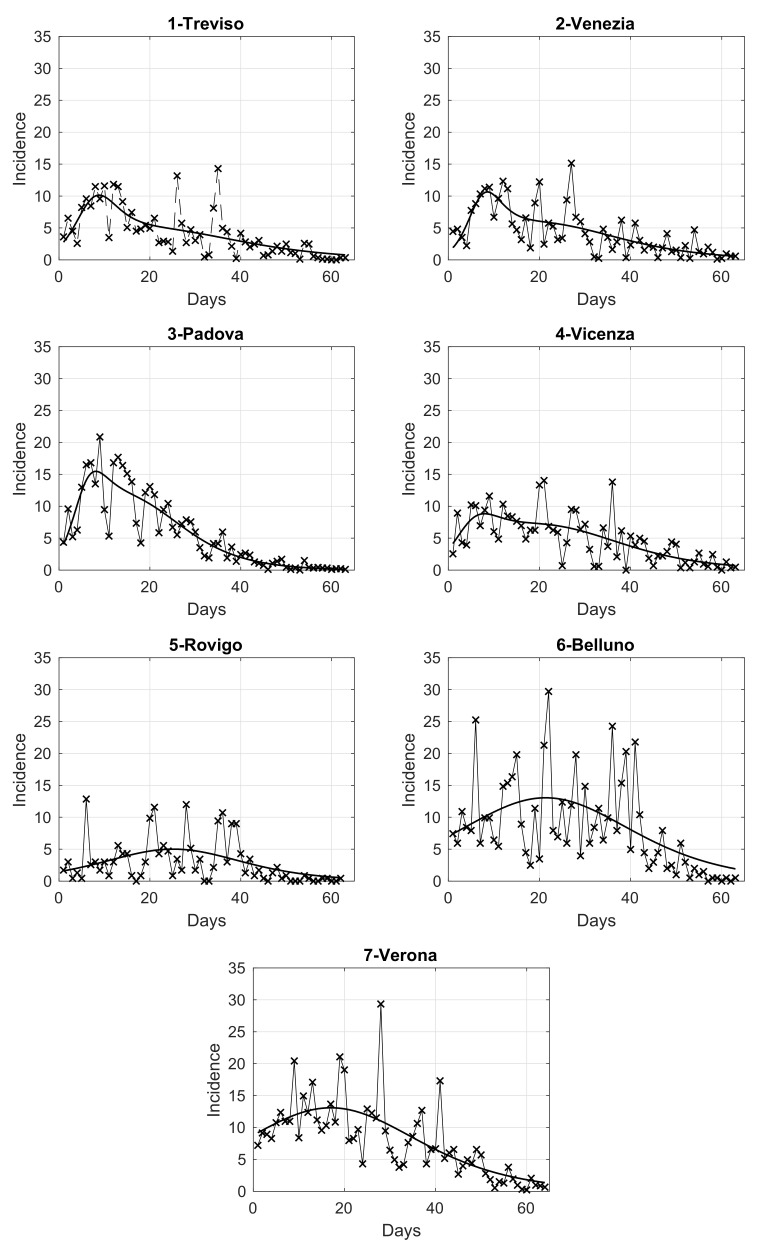
Daily SARS-CoV-2 incidence in the seven provinces of Veneto, relative to the period from 12 March to 15 May 2020, normalized by the corresponding population at 31 December 2019, referred to 100,000 inhabitants. The continuous curve overlapped to the data is the best fit given by the extended logistic model.

In order to analyze the temporal evolution of the intensity in the seven provinces of the Veneto region, we compute the daily number of SARS-CoV-2 positive cases in each of them, normalized by the corresponding population size. The results are shown in panels from 1 to 7 of Figure 3, where we also overlap a fit with the adopted extended logistic model, as it gives the best adaptation to data among some different models considered. By looking at these figures, we notice that some provinces share a similar pattern. This is further investigated below through a clustering analysis.

**Figure 4:**
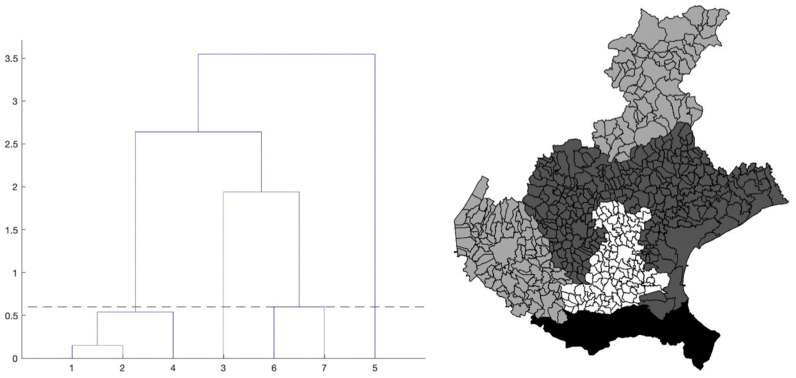
Left: dendrogram from the hierarchical clustering analysis applied to the pairs (maximum value, maximum location) of the estimated curves of the daily SARS-CoV-2 normalized incidence of the seven Veneto provinces, shown in Figure 3. The numbers on the *x*-axis correspond to the province numbering in Figure 2, right panel. The numbers on the *y*-axis are instead the square root of the difference between the summation over the k clusters of the within cluster sum of squares, and the same quantity for k + 1 clusters, multiplied by √2. Right: provinces of the Veneto region with their municipalities, grouped according to the hierarchical clustering partition. The four province clusters identified are: cluster1 = {1-Treviso, 2-Venice, 4-Vicenza}; cluster2 = {3-Padua}; cluster3 = {6-Belluno, 7-Verona}; cluster4 = {5-Rovigo}. The greyscale intensity increases with the mean of the maximum value of the estimated curves in Figure 3, among the provinces belonging to the same cluster.

The chosen algorithm is hierarchical clustering, introduced in Section 2. The dendrogram that we obtain is shown in Figure 4, left panel. To find an optimal value k_opt_ for the number of clusters, we use the elbow criterion slightly modified (described in the same section), which gives k_opt_ = 4 clusters. By cutting the tree in the dendrogram of Figure 4 (left panel) by the suitable horizontal line, we identify the elements of each of these four clusters. Precisely, we obtain: cluster1 = {1-Treviso, 2-Venice, 4-Vicenza}; cluster2 = {3-Padua}; cluster3 = {6-Belluno, 7-Verona}; cluster4 = {5-Rovigo} (the province numbers are the same as in Figure 2, right panel).

In the right panel of Figure 4, we show the map of the four identified clusters of Veneto provinces. The greyscale intensity increases with the mean among the provinces in the same cluster of the maximum value of the estimated curves in Figure 3. We see that one of the clusters is composed of three connected neighboring provinces (Vicenza, Treviso and Venice), as the intuition suggests. Two clusters contain instead only one province: Rovigo and Padua. They respectively correspond exactly to the lowest and highest maximum values of the curves. The curves of Treviso, Venice and Vicenza reached a peak of medium level approximately equal to 10 new cases per day for 100,000 inhabitants, around 20 March, while the lockdown at national level started on 12 March. We notice that the three considered provinces are neighbors. The curve of Padua also reaches the peak 8 days after the start of the lockdown, but the level was higher (about 15 new cases per day for 100,000 inhabitants). Differently, the province of Rovigo shows a peak of low value, around 5 new cases per day for 100,000 inhabitants, reached after a bit more than three weeks from the start of the national lockdown. The curves of Belluno and Verona share an intermediate peak value around 3 and 2.5 weeks since the start of the lockdown, respectively. Surprisingly, we also have the interesting result of one cluster containing two provinces (Belluno and Verona) whose distance between their main municipalities is ≈120 km as the crow flies and ≈210 km by highway.

The comparison between right panels of Figure 2 and Figure 4 shows that the maps share a similar pattern. In both the cases, Rovigo is the darkest province. Then, increasing the normalized incidence, we find the three provinces of Treviso, Venice and Vicenza with very similar values, and they belong to the same cluster. Still increasing the normalized incidence, we then find Padua, and after it, the pair Verona and Belluno. The same pattern can be seen in the map of clusters, but inverting the position of Verona and Belluno with respect to Padua.

To further strengthen the results of the above pattern, we perform the Wilcoxon Mann-Whitney test. Specifically, we compare the mean incidence of provinces, both within and between clusters. The test finds no significant difference between the mean incidence of provinces belonging to the same cluster (when it is composed by several provinces). Instead, when comparing the mean incidence of provinces belonging to different clusters, we get a significant difference for all pairs, but Padua vs. the Belluno-Verona cluster. Nevertheless, the mean value in the latter is still larger than the one for Padua.

**Figure 5:**
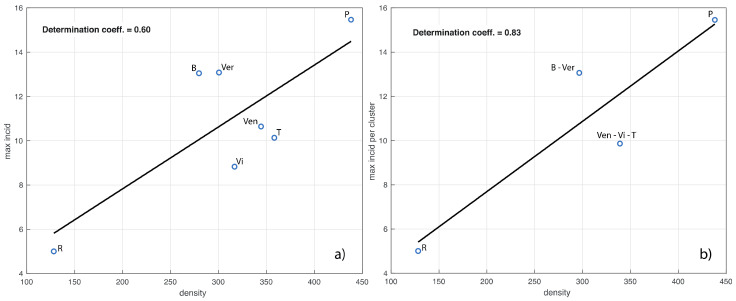
In panel (**a**) we show the linear fit of the maximum value of the province incidence curves in Figure 3 vs. the corresponding population density. In panel (**b**), the provinces are grouped according to the clusters shown in the right panel of Figure 4. The determination coefficients are also shown. The acronyms B, Ver, Ven, T, Vi, P and R stand for Belluno, Verona, Venezia, Treviso, Vicenza, Padua and Rovigo, respectively.

We now investigate the relationship between population density and maximum value of the incidence in each province, that is, the value of the maximum of the curves in Figure 3. In the left panel of Figure 5 we show the corresponding linear fit, which has a determination coefficient of 0.6. We see that the values of both variables for the provinces are close within the clusters of Figure 4. According to this observation, in the right panel of Figure 5 we also show the linear fit for the provinces grouped according to these clusters (determination coefficient of 0.83).

**Figure 6:**
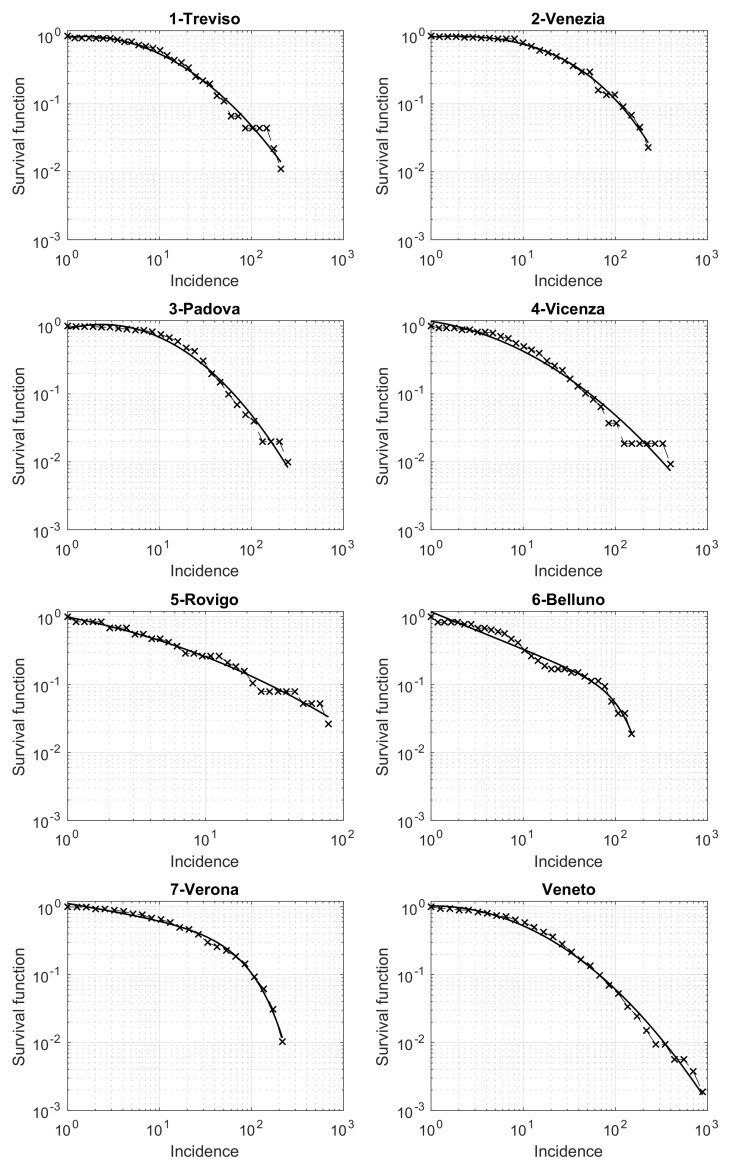
Survival function of the Veneto municipalities incidence in the period from 12 March to 15 May 2020, in a loglog scale. The best fit by the tapered Pareto model is shown by a continuous black line.

Last, but not least, we now present a relevant result on the survival function of the cumulative incidence for the different municipalities. The analysis is performed both for the entire Veneto region and separately for the seven provinces. In Figure 6, we show the results obtained in a loglog scale. The qualitative trend in each panel of the figure seems exactly the same as the one appearing in several different applied contexts, such as seismology, social science, etc. [20,21,22,23]. The key element that explains this pattern is the presence of an underline network with nodes and edges differently contextualized. In our case, nodes are patients who tested positive for SARS-CoV-2, while the edges connect pairs of individuals related by infection. Modeling the edges by the suitable random model, the survival distribution is theoretically a power law, which turns to be linear in a loglog scale [24]. However, some progressive deviations from this law are observed for high values of the variable considered (here the cumulative incidence). These deviations further decrease the values of the survival function, due to some external limiting mechanisms influencing the process. For example, in seismology, such an external factor is the finiteness of the deformational energy, which impedes very strong earthquakes to occur in a given area just ruptured by another strong shock [22,25]. In our context, this limitation could be related to the limited number of contacts that a person may have in a given time-interval. A well-known theoretical model to cope with this trend is the so-called tapered Pareto law [18,26] which, also in our case, provides a very good fit for the data, as shown in Figure 6 (black continuous lines).

## 4. Discussion

Zoonotic and arthropod-borne viruses newly emerging as a consequence of globalized traveling, climate change, intensive animal breeding, deforestation and urbanization are posing a great public health threat to our societies [27,28,29,30]. COVID-19 is a paradigm of an emerging zoonotic disease caused by the new SARS-CoV-2 coronavirus, that in a few months has swept the world, catching nations and health systems totally unaware and unprepared to stand a formidable challenge [31,32]. Notwithstanding, the great achievements already attained in dissecting the virus structure and biology may help to provide preventive and therapeutic measures in the near future, only availability of data elaborated through modeling systems with the capability of provisionally anticipating pandemic evolution could be of immediate help to decision-making health authorities.

With the aim of contributing to this effort, here we perform a spatiotemporal analysis of incidence cases occurring in Veneto region during the period of the Italian national lockdown, that is, from 12 March to 15 May 2020. Spatial signatures are related to 563 municipalities and 7 provinces of a region with almost 5 million inhabitants, one of the first Italian sites of the COVID-19 outbreak.

Our results first outline a clear clustering phenomenon of incident cases in our density map. In fact, provinces like Venice, Treviso and Vicenza cluster together, a condition that might relate these provinces sharing a spatial proximity with each other due to roads networks and good transportation. Padua, by the way, although being geographically in the middle of Veneto, stands alone with the highest incidence of the whole region. This anomaly could reflect the fact that a few municipalities surrounding the city of Padua, like Vò and Limena, were heavily involved with SARS-CoV-2 spreading and Padua city hospital with a number of elderly shelter houses were also relevant contagion loci. Then, the areas involved were efficiently isolated from the rest of the region. On the other hand, it is quite difficult to explain why two spatially distant provinces like Verona and Belluno belong to the same cluster. In fact, there is no apparent networking of social and public relations linking them. One possible explanation could be that Verona presented a nosocomial outbreak, and Belluno had an intense flare up of COVID-19 cases originally starting in the mountain municipalities close to an east Tirol outbreak happened close in time. Rovigo province, instead, although neighbor to Padua, is a cluster on its own, a phenomenon likely due to a low density population spread out in a wide agricultural environment. The result we obtain for clustering can be compared to that reported in the paper [7]. In fact, in Figure 4 of that paper, we can observe that the weights of the “epidemic-between” (inter-provinces) component of the provinces of Treviso and Venezia are both large, and we remark that they belong to one of the clusters we identified. Still looking at the epidemic-between map, the only other group sharing weights of similar strength are Parma, Bergamo and Cremona, which is surprising to us. In fact, we would expect a higher number of cases of neighboring provinces sharing a similar value of weights instead of isolated provinces. The method based on the parametric model we use to identify clusters can also be compared to the other methodologies for spatial clustering analysis of COVID-19 data typically used in the literature, for example in [10], based on the global Moran’s *I* measure and the local LISA indicators. Despite these statistics are more general than our parametric approach, we think that the latter is more robust for our case of study, as here we can explicitly model the daily incidence with a suitable extended logistic model depending only on a few parameters.

We also notice that the provinces inside each cluster we obtain seem to have very close population density values. We can also see that population density is linearly related to the strength of the pandemic, as given by the peak level of the normalized incidence curve. This then suggests that we consider the population density as a relevant variable when developing measures to control the pandemic. Among other factors that could foster or slow down the pandemic, there are those of climatic type, as proposed in several papers in the literature, see for example [33,34]. Since our work mainly concerns the spatial characterization of the COVID-19 incidence curve, we need to investigate the variation of relevant climatic parameters between the different provinces for each of the three months in the temporal window considered. Let us then focus on the temperature, which is the parameter that most likely could play a role in our case. Apart from the province of Belluno, the variation coefficients of temperature for March, April and May were 0.06, zero and zero, respectively. Therefore, it is unlikely that this climatic factor could explain the results obtained in our paper. Paradoxically, the province of Belluno, showing significant difference in each of the three months with respect to Rovigo, belongs to the latter’s same cluster.

Another relevant result is obtained when looking at the municipalities incidence’s survival function. For small values of the incidence, the function is properly described by a power law, as already seen in several applied fields such as seismology, economy and social sciences. This reveals the influence on the analyzed phenomenon of an underlying network. In this context, the network consists of the set of individuals, whose pairwise relationships represent the paths of infection. However, similarly to other applied fields, some deviations from this law appear in the survival function when we increase the value of the incidence. Nevertheless, we are able to globally describe this function by the well-known tapered Pareto model. This is observed for both each of the seven Veneto provinces and the entire region. Further investigations are needed for a larger number of provinces in Italy and abroad, to establish some dependence of the model’s parameters on some relevant quantities of interest for public health. The survival function for the COVID-19 incidence is also used by [35] in the USA counties. However, these Authors are only able to fit the final part of the survival function by a power law model. Additional investigations could help to compare their approach to ours.

The results obtained above about the clustering and the survival function contain helpful information, potentially useable when implementing preventive or containment actions. The presence of clusters provides evidence of a common diffusion of pandemic within each of them. Therefore, it makes sense to apply measures to limit pandemic diffusion homogeneously within those clusters where there is an expansion of the pandemic, making the measures more effective and minimizing the total socio-economical cost. Some quantitative information about the underlying model for the survival function of the incidence could be useful to understand the characteristics of the network through which the epidemic diffusion happens, e.g., topology. Therefore, special measures, for example related to the public transportation system, could be designed in order to avoid the presence of topologies corresponding to stronger spread.

## 5. Conclusions

In this paper, we provide methodological tools that can be used to study the spatiotemporal dynamics of SARS-CoV-2 pandemic from data of COVID-19 patients. We first deal with the partition of a spatial region of interest into separated groups based on the similarity of the pattern of diffusion, as quantified by the daily positive patients’ incidence curve. We then propose a model for the survival function of the incidence. We apply the proposed method to the Veneto region data, during the Italian lockdown. However, the proposed spatiotemporal analysis of the epidemic evolution can be applied in general in other geographical regions and periods. The detailed mathematical analysis we present, adequately corroborated with the analysis of additional data on mobility, environmental model, fatality rate, hospital admissions, intensive care unit bed availability, occupancy and exceed [36], could address the more critical areas where to invest health and medical resources to significantly contain contagion spread. In particular, it could be of interest to see if the partition obtained is the same as the one that could be obtained based on mobility data [37]. Our analysis might aid in adopting strategic decisions regarding restrictions of mobility and gatherings, deployment of public transportation, access to shops and stores, provision of medical and nursing staff and supplies. Finally, it may also impact on other public health measures, such as containment and quarantine or tools to adopt for testing and screening that may be best suited to particular local conditions.

Future work involves the application of the proposed methodology to several datasets, which we think could be useful, not only for studying the evolution of the current pandemic, as well as that of others, but also to implement effective measures to limit the virus diffusion. Extensive application of the model for the survival function of the incidence, to both real and simulated data, should be performed in order to discover the possible relation between some characteristics of the network, e.g., topology, and qualitative or quantitative properties of the chosen model.

## Data Availability

The data presented in this study are available on request from the corresponding author. The data are not publicly available due to privacy reasons.
